# Secondary choledocholithiasis caused by fish bones: a case report and review of the literature

**DOI:** 10.1186/s13256-025-05705-1

**Published:** 2025-12-01

**Authors:** Luyao Zhang, Jingbang Huang, Qinyue Liu, Chaonong Cai, Peiping Li

**Affiliations:** 1 Department of Hepatobiliary Surgery and Liver Transplantation, The Fifth Affiliated Hospital of Sun Yat-Sen University, 52 Mei Hua East Road, Zhuhai, Guangdong China; 2 Centro Hospitalar Conde de São Januário, Estrada do Visconde de São Januário, Macau, China

**Keywords:** Acute cholangitis, Choledocholithiasis, Common bile duct, Foreign body

## Abstract

**Background:**

Foreign body in the common bile duct can act as a nidus for stone formation, leading to secondary choledocholithiasis. Currently there are very few reviews concerning biliary stones caused by foreign bodies; this article may offer some help for relevant situations.

**Case presentation:**

We retrospectively analyzed the case of an 89-year-old Chinese (Han ethnicity, Asian) male patient with secondary choledocholithiasis caused by fish bones. We also present a review of the literature regarding choledocholithiasis caused by foreign bodies using “foreign body case” and “common bile duct stone” as the search terms in the PubMed database. The analysis of patient demographics, foreign body origins, clinical presentations, treatments, and outcomes may provide some suggestions for the management of those specific biliary foreign body cases.

**Conclusion:**

Even years after cholecystectomy, endoscopic retrograde cholangiopancreatography, or foreign body ingestion, biliary complications secondary to foreign body migration should still be considered. The clinical presentation of most biliary foreign bodies is similar to choledocholithiasis. endoscopic retrograde cholangiopancreatography can serve as the preferred treatment modality. When endoscopic retrograde cholangiopancreatography fails, repeating endoscopic retrograde cholangiopancreatography or conversion to surgery should be considered.

**Supplementary Information:**

The online version contains supplementary material available at 10.1186/s13256-025-05705-1.

## Background

The incidence of gallstones is about 15% [1]. Among patients with cholelithiasis, 10–20% are found to have concomitant choledocholithiasis [2, 3]. Choledocholithiasis is a common digestive system disease. When causing biliary stenosis, it can lead to acute cholangitis, a life-threatening complication [4]. Mortality risk is high if the condition is not treated with antibiotic therapy and biliary pressure is not immediately reduced using appropriate methods [5].

There are many factors contributing to the formation of choledocholithiasis. In the presence of conditions such as biliary tract infection, bile stasis, or biliary ascariasis, the calculus in the common bile duct (CBD) may originate from the bile duct system, known as the primary stones [6]. The stones may also have been caused by the decline of stones in the gallbladder, and therefore are called migrating stones or secondary stones [7].

Secondary choledocholithiasis is usually considered as an extra-cystic complication of gallbladder stones, but there are a few exceptions, such as secondary choledocholithiasis caused by foreign bodies in the CBD [8]. Choledocholithiasis caused by foreign bodies is extremely rare, with few related reports available to date. Studies have shown that the majority of retained stones present within 2–3 years of surgery. Presentation later than that is usually thought to be secondary to migratory surgical clips as it acts as a nidus for stone formation [9]. The clinical presentation of biliary foreign bodies is usually similar to choledocholithiasis, including symptoms such as abdominal pain, fever, and jaundice. However, choledocholithiasis caused by biliary foreign bodies may exhibit some specific clinical symptoms depending on the nature of the foreign body, such as nausea, vomiting, and melena [10, 11]. Therefore, for patients presenting with typical symptoms years after cholecystectomy, endoscopic retrograde cholangiopancreatography (ERCP), firearm injury, foreign body ingestion, or other specific medical histories, the diagnostic possibility of biliary complications due to foreign bodies should also be considered.

The primary treatments for choledocholithiasis caused by foreign bodies are surgery and ERCP, while a relatively small proportion of people chose percutaneous transhepatic biliary drainage [12] or conservative treatment [13, 14]. Currently, there are very few reviews concerning biliary stones caused by foreign bodies. Here we report a case of secondary choledocholithiasis with acute cholangitis caused by fish bones, and review all case reports of choledocholithiasis caused by foreign bodies. The analysis of patient demographics, foreign body origins, clinical presentations, treatments, and outcomes may provide some suggestions for the management of those specific biliary foreign body cases.

## Case presentation

An 89-year-old Chinese (Han ethnicity, Asian) male patient presented with a 10-day history of abdominal pain and fever was referred to our department. The patient had undergone a subtotal gastrectomy for upper gastrointestinal bleeding 15 years ago (specific details unknown). There was no tenderness in his abdomen during admission.

**Fig. 1:**
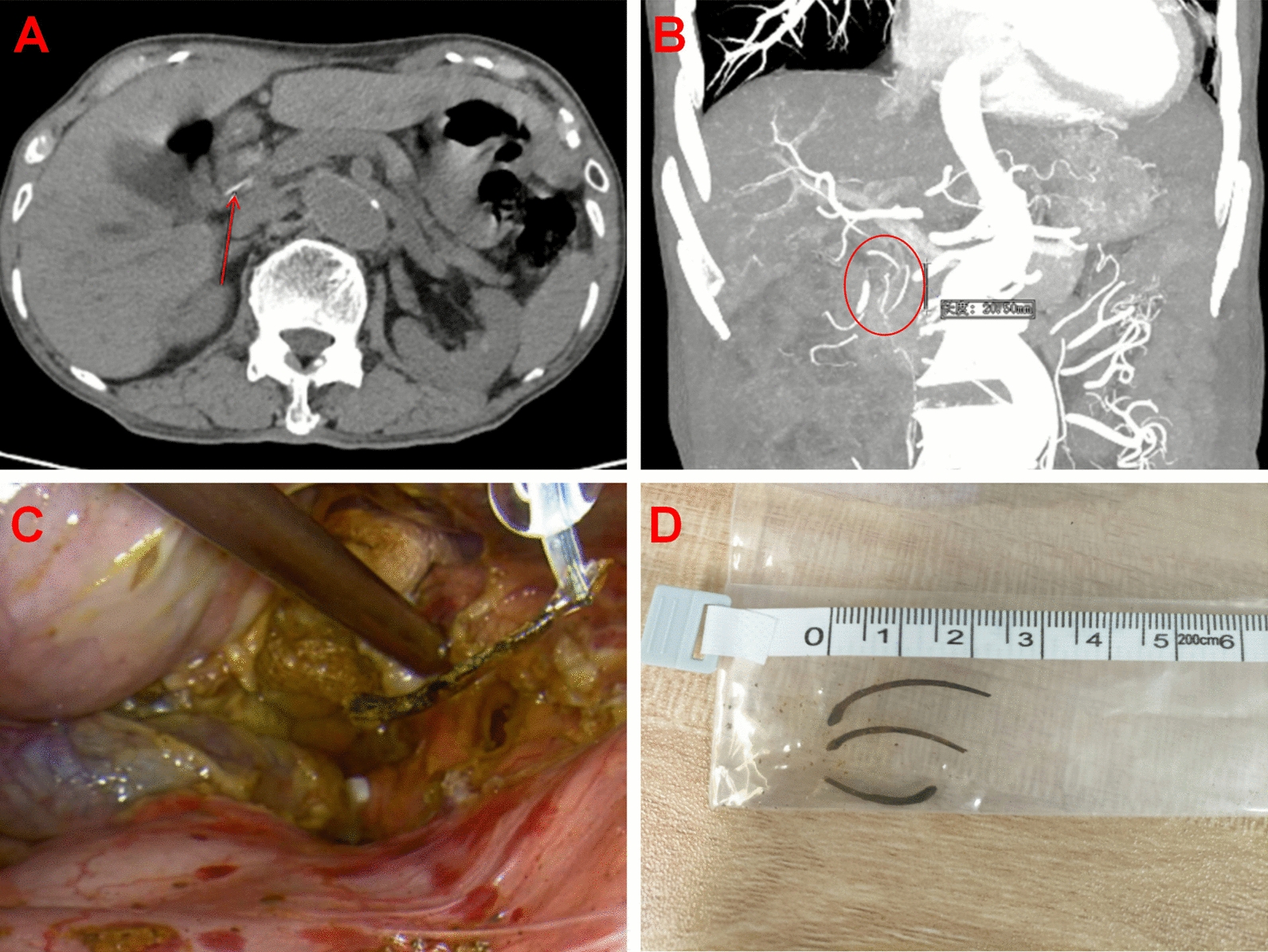
Exploration and truth of the long high-density shadows. **A** Axial enhanced computed tomography scan image reveals several strips of hyperdense (red arrow) inside common bile duct. **B** Coronal enhanced computed tomography view shows the three long high-density shadows (red circle) within common bile duct. **C** Fish bones were unexpectedly discovered and retrieved during common bile duct exploration. **D** The surgical specimen photo showed three sharp linear fish bones

Blood tests revealed impaired liver function, with Total Bilirubin (TBIL) 11 umol/L and Aspartate Aminotransferase (AST) 59.1 umol/L. Inflammatory markers were elevated, including WBC 10.3 × 10^9^/L, Neutrophils (NEU) 80.7%, procalcitonin (PCT) 0.75 ng/ml (range, 0–0.5), and C-reactive protein (CRP) 44.73 mg/L. Carbohydrate Antigen 19-9 (CA19-9) was elevated to 390 U/ml (range, 0–30). Abdominal enhanced computed tomography (CT) showed gallstones, significant dilation of the intrahepatic and extrahepatic bile ducts, and three linear high-density shadows within common bile duct (CBD) (Fig. 1AB). Notably, the patient had no history of stent implantation. We diagnosed cholelithiasis with acute cholecystitis and choledocholithiasis with acute cholangitis initially. However, the nature of the high-density shadows within CBD remained unknown.

Under general anesthesia, LC and CBD exploration was performed on 21 August 2023 (Video 1). Intraoperatively, the gallbladder was significantly enlarged, measuring approximately 10 × 4 × 3 cm, with congestion and edema on the surface. CBD was severely adhered to the ventral side, markedly dilated, with a diameter of approximately 15 mm. Intraoperative choledochoscope revealed multiple stones within CBD, along with several fish bones encased in stones, all of which were successfully removed, with the longest measuring approximately 23 mm (Fig. 1CD). A T-tube was left in place in the CBD after stone extraction.

Further inquiry into the patient’s history revealed tooth loss, non-use of dentures, and preference for fish consumption. The patient was discharged on the third day postoperatively. The T-tube was removed 7 weeks after surgery, and the patient showed good recovery through a telephone follow-up 1 year after surgery.

## Literature review

Hidden foreign body in CBD is rare and may lead to complications that include secondary choledocholithiasis. Most cases were reported as case reports. This review is based on the results of searches carried out in the PubMed database. The search terms used were “foreign body case” and “common bile duct stone”. Relevant articles were retrieved up to October 2024. We specified a protocol for the inclusion of literature: (1) foreign body does not belong to the human body or is not a parasite; (2) foreign body causes biliary stones; and (3) foreign body was hidden in CBD.

All studies that contained material applicable to the topic were considered. Retrieved manuscripts were reviewed by the authors, and the data were extracted following a standardized protocol. Data was analyzed using IBM SPSS software, version 25.0.

## Results

**Fig. 2:**
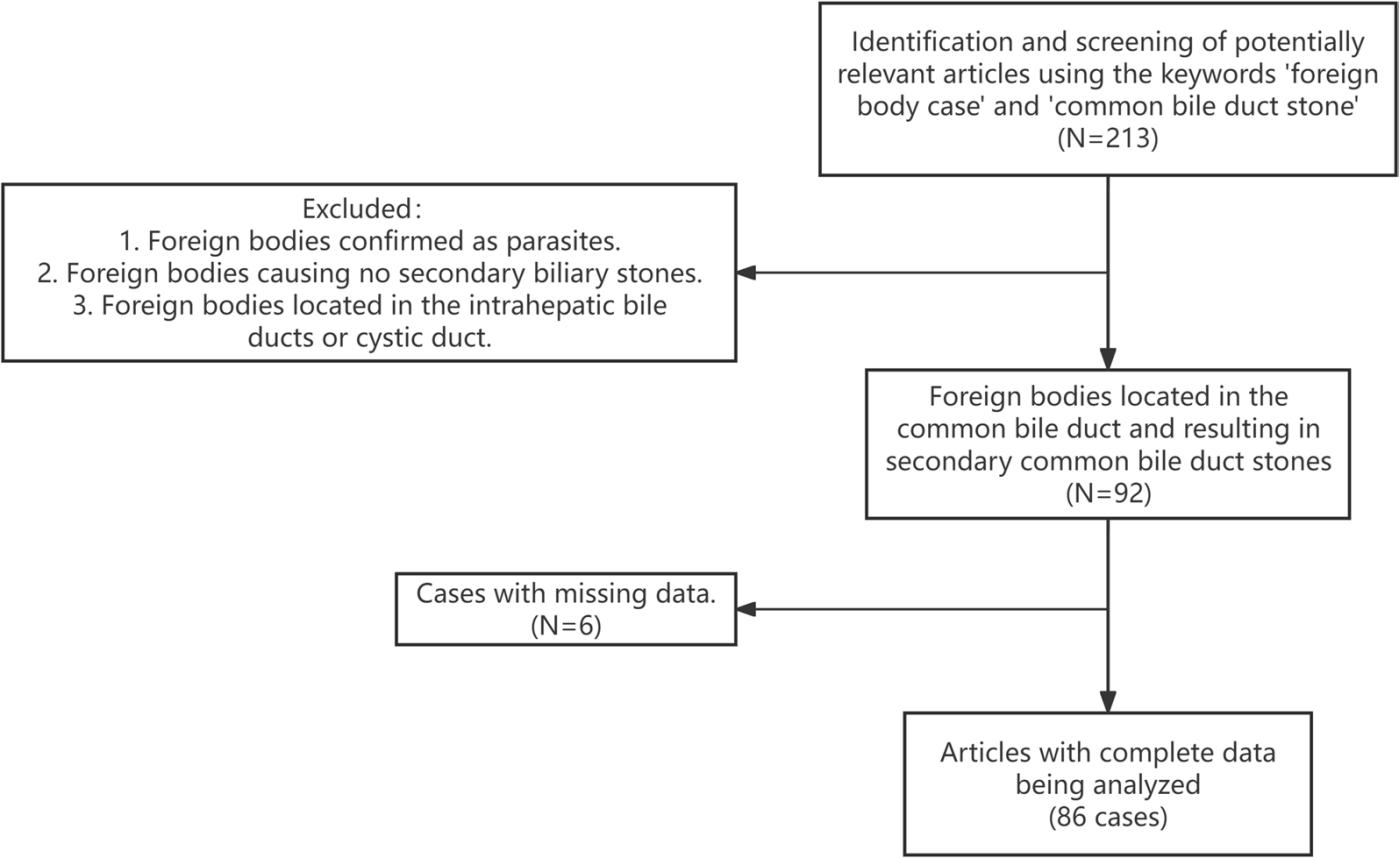
Flow chart of study selection process

A total of 213 papers were identified, but details for only 86 cases were available for the present study [12–84] (Fig. 2). The mean age at diagnosis for stones caused by foreign bodies was 62.9 years (range 19–88 years). There was no statistical significance between the genders (male 45.3% versus female 54.7%).

**Fig. 3:**
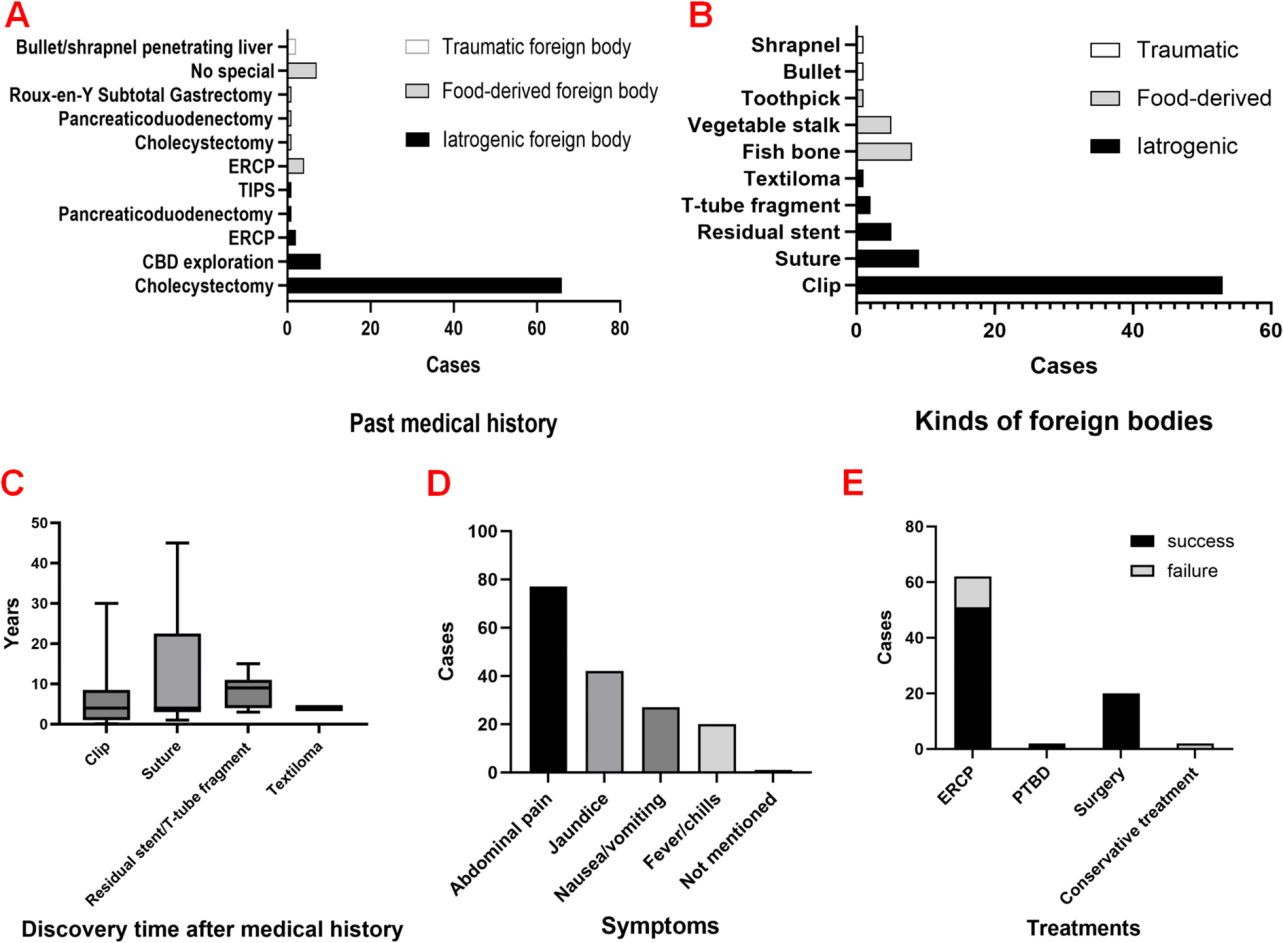
Description of choledocholithiasis caused by common bile duct foreign bodies in the literature review. **A** Patients with iatrogenic foreign bodies mostly had a history of cholecystectomy, while the most common medical history of those with food-derived foreign bodies was endoscopic retrograde cholangiopancreatography. **B** The most common iatrogenic foreign body was surgical clip, whereas all food-derived foreign bodies were sharp objects such as fish bones. **C** The time interval between the last related medical history and discovery showed no statistically significant differences among various types of iatrogenic foreign bodies. **D** The most common symptoms in patients with choledocholithiasis caused by common bile duct foreign bodies were abdominal pain, followed by jaundice, nausea/vomiting, and fever/chills. **E** Primary treatment for choledocholithiasis caused by common bile duct foreign bodies was endoscopic retrograde cholangiopancreatography, followed by surgery

Details of the clinical presentations and past medical history are presented in Table 1. On the basis of the literature review there were different types of foreign bodies, and we categorized them into three types on the basis of their sources: iatrogenic (70 cases, 81.4%), food-derived (14 cases, 16.3%), and traumatic (2 cases, 2.3%) foreign bodies. Most patients with iatrogenic foreign bodies had undergone cholecystectomy (66 cases, 76.7%), followed by CBD exploration (8 cases, 9.3%) and ERCP (2 cases, 2.3%), while the most common medical history of patients with food-derived foreign bodies was ERCP (4 cases, 4.7%) (Fig. 3A).

**Table 1:** Details of clinical presentations and past medical history (*N* = 86)

Details	*N* (%)
*Clinical symptoms*	
Abdominal pain	77 (89.5%)
Jaundice	42 (48.8%)
Nausea/vomiting	27 (31.4%)
Fever/chills	20 (23.3%)
Not mentioned	1 (1.2%)
*Kinds of foreign bodies*	
(1) Iatrogenic	70 (81.4%)
Clip	53 (61.6%)
Suture	9 (10.5%)
Residual stent	5 (5.8%)
T-tube fragment	2 (2.3%)
Textiloma	1 (1.2%)
(2) Food-derived	14 (16.3%)
Fish bone	8 (9.3%)
Vegetable stalk	5 (5.8%)
Toothpick	1 (1.2%)
(3) Traumatic	2 (2.3%)
Bullet	1 (1.2%)
Shrapnel	1 (1.2%)
*Past medical history for:*	
(1) Iatrogenic foreign body	70 (81.4%)
Cholecystectomy	66 (76.7%)
CBD exploration^1^	8 (9.3%)
ERCP^2^	2 (2.3%)
Pancreaticoduodenectomy	1 (1.2%)
TIPS^3^	1 (1.2%)
(2) Food-derived foreign body	14 (16.3%)
ERCP	4 (4.7%)
Cholecystectomy	1 (1.2%)
Pancreaticoduodenectomy	1 (1.2%)
Roux-en-Y subtotal gastrectomy	1 (1.2%)
No special	7 (8.1%)
(3) Traumatic foreign body	2 (2.3%)
Bullet/shrapnel penetrating liver	2 (2.3%)
*General information*	
Sex	
Male	39 (45.3%)
Female	47 (54.7%)
Age (years)	62.9 (range 19–88)
*Treatments*	*Success/total (success rate)*
ERCP	51/62 (82.3%)
PTBD^4^	2/2 (100%)
Surgery	20/20 (100%)
Conservative treatment	0/2 (0%)

^1^Common bile duct

^2^Transjugular intrahepatic portosystemic shunt

^3^Endoscopic retrograde cholangiopancreatography

^4^Percutaneous transhepatic biliary drainage

The most common iatrogenic foreign bodies were surgical clips (53 cases, 61.6%), sutures (9 cases, 10.5%), residual stents (5 cases, 5.8%), and T-tube fragments (2 cases, 2.3%). One case involved a surgical gauze accidentally left from the previous LC, with a medical history of biliary fistula after surgery [72]. Food-derived foreign bodies (14 cases, 16.3%) were those “pike-like” objects that would pass through the human digestive tract and migrate to the CBD, which included fish bones, toothpicks, and vegetable stalks. Traumatic foreign bodies included bullet and shrapnel (2 cases, 2.3%) (Fig. 3B).

Different types of iatrogenic foreign bodies may lead to secondary CBD stones. The discovery time of iatrogenic foreign bodies (since the last related medical history) was compared in Table 2. The median discovery time of iatrogenic foreign bodies was 4 (2, 9) years, with no statistically significant differences among the various types of iatrogenic foreign bodies (*P* = 0.53) (Fig. 3C). The most common clinical presentations were abdominal pain (77 cases, 89.5%), followed by jaundice (42 cases, 48.8%), nausea/vomiting (27 cases, 31.4%), and fever/chills (20 cases, 23.3%) (Fig. 3D).

**Table 2:** The discovery time of different iatrogenic foreign bodies after the last related medical history

	Discovery time after medical history (years)	*P*-value
Iatrogenic foreign body (*N* = 70)	4 (2, 9) *	
Clip (53)	4 (1, 8)	0.530
Suture (9)	4 (3, 22.5)	
Residual stent/T-tube fragment (7)	9 (4, 11)	
Textiloma (1)	4	

*Median (interquartile range)

The main treatment methods for biliary stones caused by foreign bodies were ERCP (62 cases, 72.1%) and surgery (20 cases, 23.3%). Only a relatively small proportion of people used the methods of percutaneous transhepatic biliary drainage (PTBD) (2 cases, 2.3%) and conservative treatment (2 cases, 2.3%). We defined treatment failure as requiring a second procedure or conversion to surgery. The success rate of ERCP was 82.3% (51/62), while the two cases undergoing conservative treatment experienced treatment failure and needed conversion to ERCP (Fig. 3E).

Most patients (81 cases, 94.2%) recovered uneventfully and were perfectly well at the follow-up clinical examination, but some of them suffered long-term problems or severe complications, such as pancreatitis [38, 69], bile duct rupture/bile leakage [76], choledochoduodenal fistula [56], and septic shock [77].

## Discussion and conclusions

Most cases of biliary stones caused by foreign bodies occur in the elderly population, likely because the tissues of elderly individuals are too fragile to prevent foreign bodies from migrating. Various types of foreign bodies can all lead to secondary choledocholithiasis, with surgical clip being the most common one. The most pointed mechanism of foreign body migration is reflux from duodenum [85, 86]. Henderson *et al*. performed manometry of the greater papilla in patients with choledocholithiasis caused by foreign bodies and compared with manometry of patients with common stones. Patients with choledocholithiasis caused by foreign bodies presented greater prevalence of retrograde waves compared with patients with common stones [87], which supported duodenal reflux as a theory. While the exact pathogenesis of foreign body migration remains unknown, it is likely related to improper clip application, bile leakage, inflammation and subsequent necrosis, allowing the clips to eventually erode into the CBD [88]. Most secondary choledocholithiasis caused by iatrogenic foreign bodies occur approximately 4 years after the surgical procedure, with no significant difference in the onset time among different types of foreign bodies.

For patients with food-derived biliary foreign bodies, whenever a choledochoenteral fistula is found, it is postulated as the route for migration of the foreign body [51]. Furthermore, many cases with food-derived foreign bodies have a history of digestive tract reconstruction surgery or ERCP [24, 30, 37, 44, 50, 82], leading to the loss of Oddi sphincter function, which may allow foreign body entry into the CBD via the duodenum. Additionally, given that all previously reported food-derived foreign bodies were sharp objects (for example, toothpicks and fish bones), we hypothesize that such objects might progressively penetrate both the intestinal and biliary walls to ultimately reach the CBD.

The clinical presentation of most biliary foreign bodies is similar to choledocholithiasis. However, some patients may experience severe complications such as pancreatitis, bile duct rupture/bile leakage, choledochoduodenal fistula, and septic shock. ERCP can serve as the preferred treatment modality, with a high success rate of 82.3%. Even if stone extraction during ERCP fails, repeating ERCP or conversion to surgery can be considered.

Here we reported an unusual case of secondary choledocholithiasis caused by fish bones. The patient had a history of subtotal gastrectomy, which could have led to adhesions between the residual stomach and CBD, providing an opportunity for the fish bones to sequentially penetrate the walls of the stomach and bile duct, eventually entering the CBD. In addition, since this patient was elderly, his Oddi sphincter may have relaxed or lost function, thus there is also a possibility that the fish bones passed through the duodenal papilla and migrated into the CBD. The fish bones finally acted as cores for the formation of biliary stones.

It is worth noting that elevated CA19-9 level in this case may be attributed to secondary CBD stones causing biliary obstruction, though the possibility of malignancy cannot be excluded. Pathology of the gallbladder specimen confirmed benign lesions, and follow-up at 1 year indicated favorable recovery.

The fish bones were hidden in the CBD, with clinical manifestations mimicking choledocholithiasis, presenting a diagnostic challenge here. The only diagnostic clue in this case was the presence of linear calcifications in the preoperative CT images. However, establishing a connection between the linear calcifications and the accidentally ingested fish bones was difficult due to the fundamental isolation of the CBD from the digestive tract. In fact, it was not until we observed the fish bones during CBD exploration that we confirmed the nature of these linear calcifications. Therefore, identification and removal of the fish bones as soon as possible was crucial.

Even years after cholecystectomy, ERCP, or foreign body ingestion, biliary complications resulting from foreign body migration should still be considered. The clinical presentation of most biliary foreign bodies is similar to choledocholithiasis. ERCP can serve as the preferred treatment modality, and if it fails, repeating ERCP or conversion to surgery can be considered.

Currently, there are very few literature reviews concerning biliary stones caused by foreign bodies. This article categorizes the sources of foreign bodies leading to secondary choledocholithiasis, aiming to give some suggestions about the differential diagnosis and the options of treatment for biliary foreign bodies.

## Supplementary Information

Additional file 1.

## Data Availability

The datasets used and/or analyzed during the current study are available from the corresponding author on reasonable request.

## Abbreviations

CBD: Common bile duct
ERCP: Endoscopic retrograde cholangiopancreatography
IQR: Interquartile range
LC: Laparoscopic cholecystectomy
PTBD: Percutaneous transhepatic biliary drainage
